# The accuracy of computed tomography scans for rapid prototyping of canine skulls

**DOI:** 10.1371/journal.pone.0214123

**Published:** 2019-03-25

**Authors:** Michaela L. Comrie, Gabrielle Monteith, Alex Zur Linden, Michelle Oblak, John Phillips, Fiona M. K. James, on behalf of the Ontario Veterinary College Rapid Prototyping of Patient-specific Implants for Dogs (RaPPID) group

**Affiliations:** 1 Department Human Health and Nutritional Science, College of Biological Science, University of Guelph, Guelph, Ontario, Canada; 2 Department of Biomedical Sciences, Ontario Veterinary College, University of Guelph, Guelph, Ontario, Canada; 3 Department of Clinical Studies, Ontario Veterinary College, University of Guelph, Guelph, Ontario, Canada; 4 Centre for Advanced Manufacturing and Design Technologies, Sheridan College, Brampton, Ontario, Canada; Monash University, AUSTRALIA

## Abstract

This study’s objective was to determine the accuracy of using current computed tomography (CT) scan and software techniques for rapid prototyping by quantifying the margin of error between CT models and laser scans of canine skull specimens. Twenty canine skulls of varying morphology were selected from an anatomy collection at a veterinary school. CT scans (bone and standard algorithms) were performed for each skull, and data segmented (testing two lower threshold settings of 226HU and -650HU) into 3-D CT models. Laser scans were then performed on each skull. The CT models were compared to the corresponding laser scan to determine the error generated from the different types of CT model parameters. This error was then compared between the different types of CT models to determine the most accurate parameters. The mean errors for the 226HU CT models, both bone and standard algorithms, were not significant from zero error (*p* = 0.1076 and *p* = 0.0580, respectively). The mean errors for both -650HU CT models were significant from zero error (*p* < 0.001). Significant differences were detected between CT models for 3 CT model comparisons: Bone (*p* < 0.0001); Standard (*p* < 0.0001); and -650HU (*p* < 0.0001). For 226HU CT models, a significant difference was not detected between CT models (*p* = 0.2268). Independent of the parameters tested, the 3-D models derived from CT imaging accurately represent the real skull dimensions, with CT models differing less than 0.42 mm from the real skull dimensions. The 226HU threshold was more accurate than the -650HU threshold. For the 226HU CT models, accuracy was not dependent on the CT algorithm. For the -650 CT models, bone was more accurate than standard algorithms. Knowing the inherent error of this procedure is important for use in 3-D printing for surgical planning and medical education.

## Introduction

The standard imaging modalities available, including computed tomography (CT) scans, are limited in their ability to clearly and accurately represent three dimensional (3-D) space on two dimensional (2-D) viewing screens; such representation is helpful to understand structural complexities in medicine [1]. Therefore, in recent years, rapid prototyping technology—the transformation of a computer model to a physical 3-D model—has begun to be used in the human and veterinary medical fields in several settings [2].

In human surgery, 3-D printing research has focused on the creation of patient-specific implants and cutting guides, which have both been shown to have several advantages [3,4]. Advantages of cutting guides include a decrease in anesthesia time, a reduced need for analgesics, decreased blood loss, possibly reduced infection rates along with a decreased need for antibiotic use, reduced intraoperative fluoroscopic navigation causing a decrease in radiation exposure, and improved surgical outcome [3,4]. In the field of neurology specifically, 3-D printing has been reported for surgical planning, including for neurosurgery and cranial/orbital surgery [5], as well as for the creation of custom implants and cranial plates for patients with head injuries [5,6]. Anatomical models are utilized to shape the implant prior to surgery resulting in an improved fit of the implant [5]. Although 3-D printing has been extensively reported in human medicine, the literature has mostly been case-based, which does not necessarily extrapolate to the population.

In veterinary medicine, research is even more sparse than that of human medicine. Research in canines has recently focused on experimental models of bone tissue engineering [7–10]. Three-dimensional printing has been utilized in surgical applications including prosthetic lung implantation [11], as well as surgical guides in dental implant surgery [12], rostral mandibular reconstruction [13], and ophthalmology [14]. These few cases suggested that the 3-D models had a positive impact on veterinary medicine and should therefore be utilized in surgical planning and education for students and clients, but this data is clearly lacking in neurosurgical applications.

In 3-D printing research specifically, there is not yet a standard printing process in human or veterinary medicine that those new to the field might follow [15]. There have been several proposals made for a protocol in human medicine, including recommended CT scan guidelines which give the ideal image for creating 3-D printed models [16], a quality assurance framework for all steps of the medical 3-D printing process [17], and a description of the step-by-step instructions for the entire 3-D printing process used for patient-specific brains and skulls [18]. Unfortunately, these few proposals may not be useful overall because they are not yet validated standard protocols and do not give specific and quantifiable data or instructions that can be replicated.

Thus, although there have been descriptions of the medical applications of 3-D printing, as well as proposals for accuracy and standardization, a standard process and imaging protocol remains lacking. There are no reports of the magnitude of error that might be accumulated in the modelling process using current standard equipment and software. Clear evidence-based guidelines are needed to optimize the accuracy of the imaging modality and 3-D model used in these applications. Further, the question arises whether current standard CT scan protocols might be sufficient for accurate 3-D modelling, without developing a specific 3-D scanning protocol that may have a higher radiation dose or may only be performed in select cases. Assessing the utility of routine CT scan protocols would open the possibility of retrospective and prospective data collection such that any patient could have an accurate 3-D model made. The purpose of this study is therefore to determine the optimal 3-D digital modelling process for canine skulls, an example of complex anatomical topography, using standard medical patient CT scan image acquisition protocols. Clarifying the 3-D digitization process for routine CT images and understanding their inherent error will provide a basis for future accuracy claims in 3-D design and printing. The objective for this particular study is to quantify the error between the 3-D models generated from standard medical CT scans and gold-standard 3-D laser scans of canine skulls, by overlaying the digital images and measuring the difference, in millimeters, to determine the accuracy of 3-D printing for future applications. The hypothesis of this study is that 3-D digital skull models derived from CT imaging accurately represent the real skull dimensions.

## Materials and methods

### Sample

Twenty preserved canine skull specimens of varying morphology, and devoid of any soft tissue structures, were selected from the Veterinary Anatomy Laboratory in the Department of Biomedical Sciences at the University of Guelph. These were chosen at random with no exclusion criteria.

### Procedures

Each skull was digitized via two methods: 1) CT scanning and 2) laser scanning.

#### 1. CT scanning

**Fig 1:**
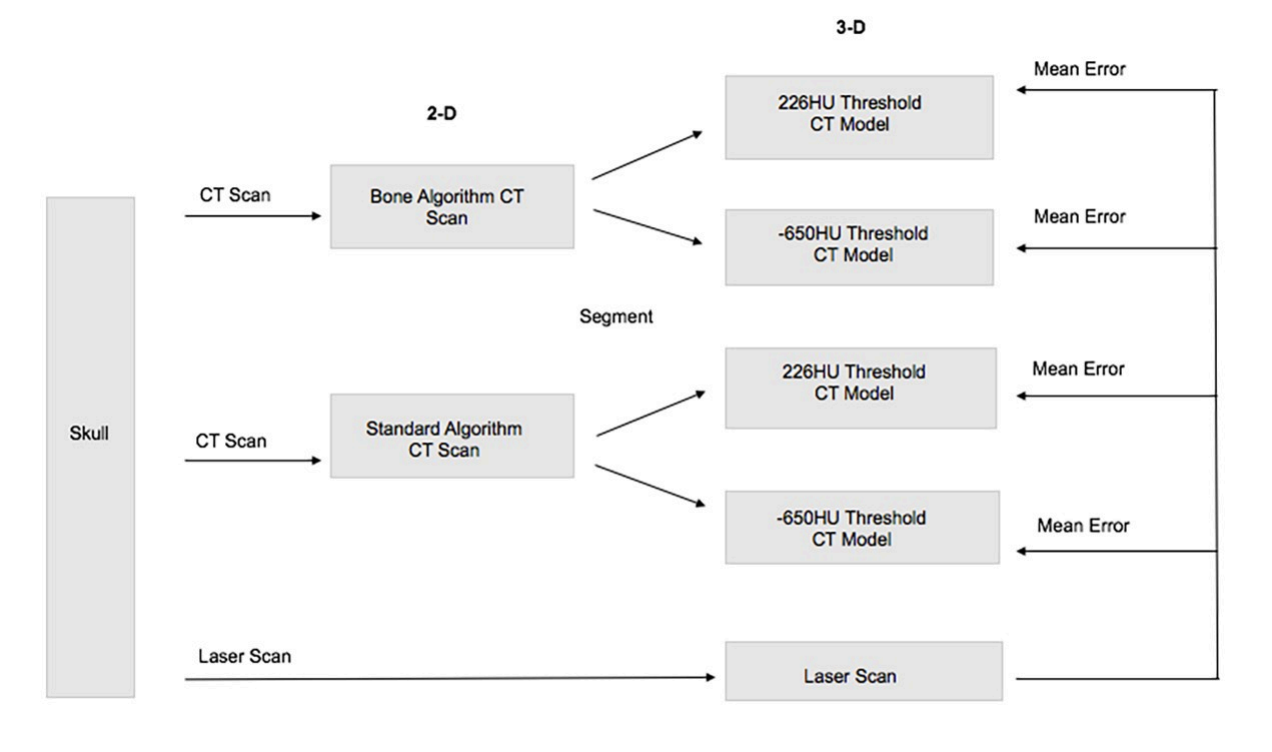
Procedure flowchart. Flowchart representing the simplified procedure for a single canine skull. This procedure was repeated for each of the 20 skulls in the sample, resulting in a total of 20 mean errors calculated for each of the four types of CT model compared to its corresponding laser scan.

CT scans for each skull were obtained using the medical imaging parameters in accordance with the Ontario Veterinary College Health Sciences Centre patient protocols. This procedure was completed using a 16-slice detector GE Brightspeed CT scanner. The raw data were acquired with a standardized protocol in helical mode, 1.0-second rotation time, 0.562:1 pitch, 120 kV and 250 mA, 25-cm collimation, 512 x 512 matrix size, 0.488 mm in plane resolution, 0.625 mm through plane resolution. The dataset underwent reconstructions using both Bone Plus and Standard algorithms. This protocol gave a total of two CT datasets per skull **(**Fig 1**)** and forty CT datasets across all skulls. The 2-D CT scans were then transformed into digital 3-D CT models using Materialise Mimics Research version 19.0, 3-D medical image processing software. This procedure was completed twice for each CT scan, via differing systematic threshold adjustments of contrast and brightness. The threshold value was represented using Hounsfield units (HU), which vary based on the tissue density, more specifically the linear attenuation coefficient. The first segmentation was done using a software default minimum threshold of 226HU, and then the *Thresholding* option was opened up to include HU to a manual minimum threshold of -650HU for the second segmentation. The first threshold value was chosen based on the default threshold set for bone tissue CT imaging in the software, while the second threshold value was selected from a published description of bone tissue window values for CT scan imaging [19]. For both segmentations, the maximum threshold varied based on the limit for contrast and brightness determined by the software for each individual CT scan. The upper skull was then separated from the mandible using the *Split Mask* option, by manually highlighting the region of interest in each slice. The articular surface of the temporomandibular joint was not included in the comparison. These slices were manually edited to ensure the separation was accurate using the *Edit Masks* option. The separate upper skull mask was then made into a 3-D CT model using the *Calculate 3-D* option. This procedure therefore created a total of four CT models per skull **(**Fig 1**)** and eighty CT models across all skulls. The CT models were then exported to Materialise 3-matic version 11.0, 3-D modelling software, and saved in stereolithography (STL) format.

#### 2. Laser scanning

The laser scans for each upper skull were obtained using a NextEngine 3D Scanner HD laser scanner. To capture all angles, a total of 2–4 scans had to be completed per skull depending on size. The laser scans were processed using Auto-Drive, with setting preferences consisting of 16 divisions, 17k (HD) points/square-inch, neutral target, macro range, and either 360 degree positioning or bracket positioning. These preferences were specified in NextEngine ScanStudio HD version 2.0.2, 3-D modelling software, and the scans were then processed using the same software. The base used to hold the skull upright for each scan was removed using the *Trim* option, and the scans for each individual skull were then combined using the *Align* option and secured using the *Fuse* option. This procedure therefore created one laser scan per skull **(**Fig 1**)** and twenty laser scans across all skulls. The laser scans were then saved in STL format.

**Fig 2:**
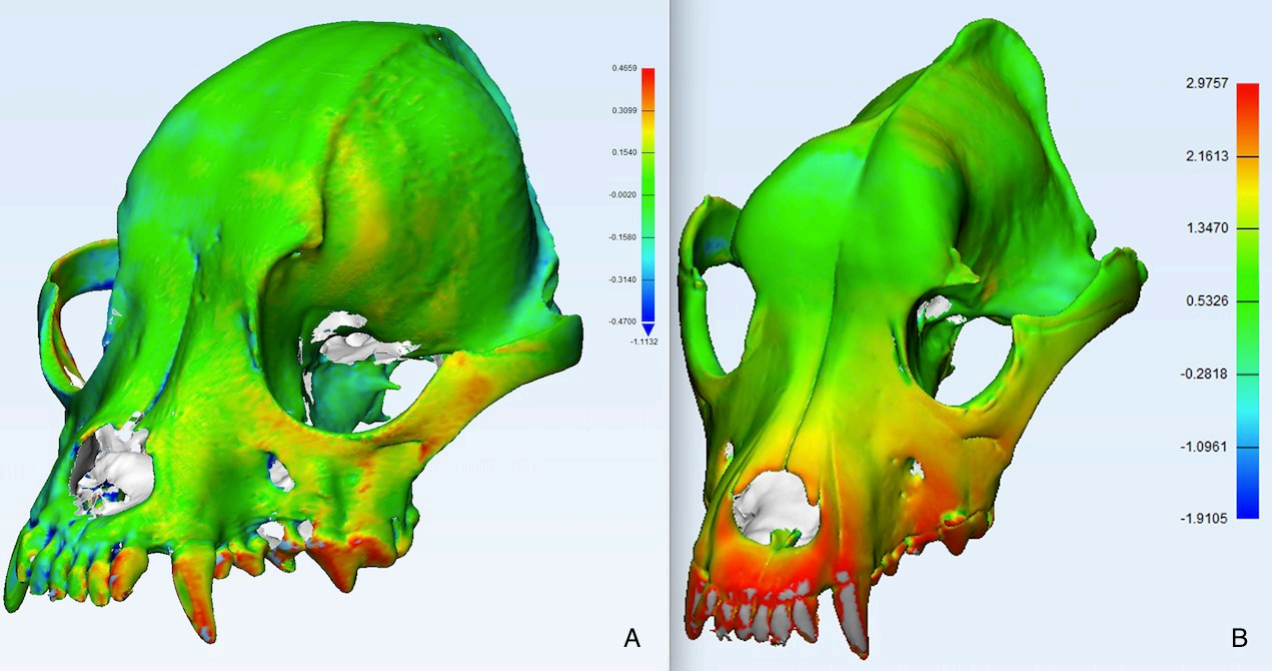
Sample error maps. Shown is an image of the aligned CT model and its corresponding laser scan for two different skulls. The colour map indicates the deviation in millimeters between the two models as determined by the software analysis. (A) Skull 20, indicating a small mean error. (B) Skull 9, indicating a relatively larger mean error.

All STL models for the eighty CT models and the twenty laser scans were imported into Materialise 3-matic version 11.0, 3-D modelling software. The laser scan represents the outside skull surface, therefore the outside surface of each CT model was highlighted using either the *Brush Mark* option or *Area Mark* option and copied to a new part. Each CT model outside surface was then aligned to its corresponding laser scan using the *Align* option, first by *N Points Registration* and then by *Global Registration*. The aligned 3-D images were then compared using a least squares fitting algorithm via the *Create Part Comparison Analysis* option and *Signed* method (Fig 2), the latter algorithm calculating the signed error from the comparison indicating whether the target entity was inside (negative error) or outside (positive error) the selected entity. The output for this comparison determined the mean error between the CT models and the laser scan, measured in millimeters. This procedure was repeated across all skulls for each of the four types of CT models compared to its corresponding laser scan **(**Fig 1**)**.

### Statistical analysis

Overall mean error ± standard deviation (SD) and 95% confidence interval were calculated across all skulls for each of the four types of CT models. In addition, a 2-sided tolerance limit was calculated across all skulls for each of the four types of CT models to determine the error range in which 99% of the population of CT skull models would fall into with 95% confidence.

Student’s t-tests were used to determine if the CT model mean errors were significantly different from zero error, indicating a significant difference from the laser scan or real skull dimensions. The level of significance was set at 0.05.

Wilcoxon signed-rank tests were used to determine if there was a significant difference between the different CT model parameters: 1) Bone CT models: 226HU threshold compared to -650HU threshold; 2) Standard CT models: 226HU threshold compared to -650HU threshold; 3) 226HU threshold CT models: bone algorithm compared to standard algorithm; 4) -650HU threshold CT models: bone algorithm compared to standard algorithm. The level of significance was set at 0.05.

In addition, a trend in the data appeared which suggested that larger skulls tended to have larger mean errors and standard deviations. Nineteen skulls were weighed without their mandibles. One skull (specimen 2) could not be accurately weighed due to the fact that the mandible and C1 were permanently mounted to it. To examine this trend, Pearson correlation coefficients were calculated between average skull weight and both the mean error and average SD across all skulls for each of the four types of CT models. The Spearman correlation was used to determine if there was an effect of skull weight measured in grams with the coefficient of variation (CV) for the four types of CT models.

All analyses were performed by use of Base SAS version 9.4 statistical analysis software.

## Results

Of the 20 skulls included in the study, the breed was known for 14 (6 were of indeterminate, or mixed, breed). Breeds were Pomeranian, Boston Terrier, Dalmatian, Beagle, Dachshund, Collie, Chow Chow, Boxer, Bullmastiff, Pit Bull, Great Dane, Newfoundland, Irish Wolfhound, and Saint Bernard. The 19 weighed skulls ranged in weight from 22g to 430.7g, with mean 178.98g and standard deviation 114.25.

The CT scanner had an in-plane resolution of 0.488mm and a through-plane resolution of 0.625mm. To detect differences (errors) of this size would require 4 skulls with 93% and 99% power respectively. The Pearson correlation test had 83% power with 20 skulls to detect *r* = 0.6.

**Fig 3:**
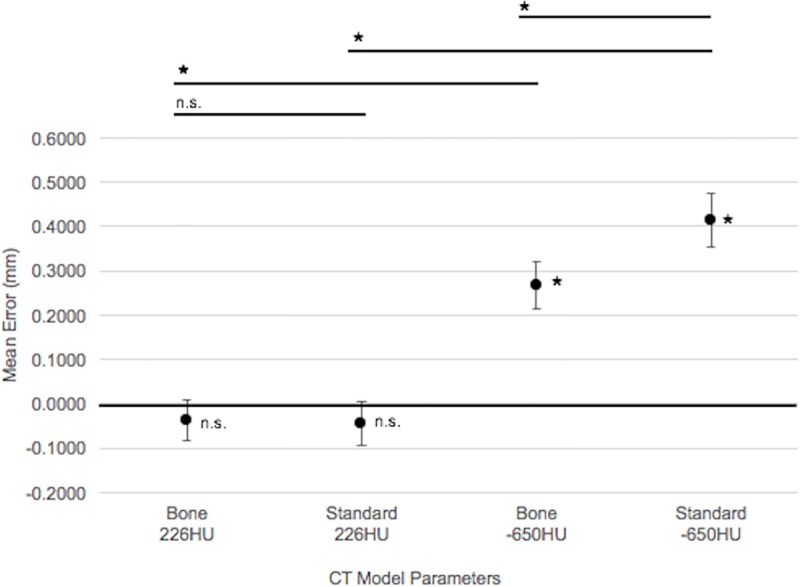
Graphical representation of the overall mean error for each of the four types of CT model compared to its corresponding laser scan. Error bars represent the 95% confidence interval around the mean. Between-group comparisons are visually represented above the graph. * *p* < 0.0001 representing a significant difference between the mean error and zero error, and representing a significant difference detected between the mean errors for comparisons 1) bone CT models, 2) standard CT models, and 4) -650HU threshold CT models. n.s. = Not significant.

Overall mean error and variance data were calculated for each of the four types of CT models as reported in Table 1. Detection of a significant difference between the overall CT model mean error and zero error varied depending on the threshold of the CT model (Fig 3). The mean errors calculated for both 226HU threshold CT models using the bone algorithm and the standard algorithm were not statistically significant (*p* = 0.1076 and *p* = 0.0580, respectively). In contrast, the mean error calculated for both -650HU threshold CT models using the bone algorithm and the standard algorithm were statistically significant (*p* < 0.0001).

**Table 1:** Summary statistics for the CT model parameters tested.

CT Model	Mean±SD (mm)	CI (95%) (mm)	TL (99% with 95%) (mm)	Significance (α = 0.05)
Bone 226HU	-0.0380 ± 0.1005	-0.0850, 0.0091	-0.4020, 0.3260	0.1076
Standard 226HU	-0.0441 ± 0.1071	-0.0985, 0.0018	-0.4360, 0.3400	0.0580
Bone -650HU	0.2671 ± 0.1137	0.2139, 0.3203	-0.1440, 0.6790	<0.0001*
Standard -650HU	0.4139 ± 0.1284	0.3538, 0.4740	-0.0511, 0.8789	<0.0001*

SD = Standard deviation. CI = Confidence interval. TL = Tolerance limit.

* significant difference from zero error.

Significant differences were detected for three of the four CT model (mean ± SD) comparisons (Fig 3). For comparison 1) bone CT models: the difference between the 226HU threshold (-0.0380mm ± 0.1005mm) and the -650HU threshold (0.2671mm ± 0.1137mm) was significant (*p* < 0.0001). For comparison 2) standard CT models: the difference between the 226HU threshold (-0.0441mm ± 0.1071mm) and the -650HU threshold (0.4139mm ± 0.1284mm) was significant (*p* < 0.0001). For comparison 4) -650HU threshold CT models: the difference between the bone algorithm (0.2671mm ± 0.1137mm) and the standard algorithm (0.4139mm ± 0.1284mm) was significant (*p* < 0.0001). In contrast to these three comparisons, for comparison 3) 226HU threshold CT models: the difference between the bone algorithm (-0.0380mm ± 0.1005mm) and the standard algorithm (-0.0441mm ± 0.1071mm) was not significant (*p* = 0.2268).

There were no significant correlations between skull weight and mean error for any of the four types of CT model. In contrast, there were positive correlations between skull weight and average SD for all of the four types of CT model: bone algorithm with 226HU threshold (*r* = 0.8674, *p* = < 0.0001); standard algorithm with 226HU threshold (*r* = 0.7828, *p* = 0.0009); bone algorithm with -650HU threshold (*r* = 0.7610, *p* = 0.0016); and standard algorithm with -650HU threshold (*r* = 0.8010, *p* = 0.0004). The Spearman correlation indicated no positive association of weight with the CV for both the standard and bone algorithms with 226HU threshold (p = 0.2684 and p = 0.5233 respectively). Both the standard and bone algorithms with 650HU threshold were significant for a positive correlation (p = 0.0027 and p = 0.00039 respectively).

## Discussion

CT scanning is the gold standard for bone imaging, and laser scanning is the gold standard for reproducing bone surfaces in exact detail. Therefore, this study was completed to quantify the error generated in 3-D digital canine skull models, compared to laser scans, by using routine veterinary patient CT scans as templates.

The data supported the original hypothesis. This study quantified the error generated when routine CT models are used for rapid prototyping and provided evidence for which commonly used CT algorithm and threshold parameters are the most accurate. Independent of the different parameters tested, the 3-D skull models derived from CT imaging accurately represent the real skull dimensions for use in a clinical veterinary setting within 0.42 mm. This error is less than the 0.488 mm CT scanner in plane resolution and also less than the 0.625 mm slice thickness [20].

CT threshold windows differ in order to view specific tissue types in better detail [19]. Independent of the image acquisition algorithm used, both types of 226HU threshold CT models had a negative mean error on average, indicating that these CT models are smaller than the laser scans. Despite this difference, the actual size difference was not significantly different from zero. This finding indicates that the 226HU threshold CT models accurately represent the laser scans, and therefore they statistically represent the real skull dimensions. In contrast, both types of -650HU threshold CT models had a positive mean error on average, indicating that these CT models are larger than the laser scans. Both of these types of CT models had a mean error that was significantly different from zero. While this result might suggest that the -650HU threshold CT models do not accurately represent the laser scans, this is a statistical difference. Clinically, these results may not have practical impact due to the very minimal error generated on the millimeter scale. All types of CT models had a mean error within 0.42 mm from the laser scans, with all confidence intervals spanning a range within 0.13 mm mean error with 95% confidence. The tolerance limits indicate that 99% of all canine skull CT models will fall within a range of only 0.93 mm mean error with 95% confidence. Clinically, these values indicate that regardless of the HU minimum threshold used, the CT models are a very accurate representation of the real skull dimensions for use in rapid prototyping for surgical planning and medical education. Moreover, the default minimum threshold set by the software (226HU) and the manual minimum threshold determined from previous literature (-650HU) [19] were statistically compared. Testing the minimum thresholds for both the bone window for the software as well as the optimal bone window as drawn from the literature determines which threshold is the most accurate for creation of the 3-D CT models. For both bone CT models and standard CT models, there was a significant difference between the mean error for the 226HU threshold compared to the -650HU threshold, with the 226HU threshold mean error being closer to the real skull dimensions in both cases. This finding indicates that the default threshold of 226HU is more accurate than the manual threshold of -650HU.

In addition, this study tested the difference between the CT image acquisition algorithm, bone versus standard, for use in rapid prototyping. The different algorithms are used for optimal viewing of specific tissue types, with the bone algorithm being optimal for visualizing bone tissue and the standard algorithm being optimal for visualizing soft tissue. For the -650 threshold CT models, there was a significant difference between the mean error for the bone algorithm compared to the standard algorithm, with the bone algorithm mean error being closer to the real skull dimensions. This was not the case for the 226HU threshold CT models, where a significant difference between the mean error for the bone algorithm compared to the standard algorithm was not detected. This indicates that for the 226HU threshold CT models, the accuracy of the procedure is not dependent on the original CT algorithm used.

This study is not free of limitations. The data suggested that skull size, and therefore canine breed, may have had an impact on the accuracy of this procedure using the 650HU threshold. It was determined that as the weight of the skulls increased, the average standard deviation also increased, which indicates that the 650HU CT models of larger skulls are less likely to produce an accurate result when used for rapid prototyping. It has been suggested that different threshold values may be required for different skull sizes [21]. This theory could explain why the larger skulls appear to be less accurate using this threshold. The data reported here are absolute errors rather than relative errors, and therefore it is possible that the relative error average standard deviation may not have showed this same trend across the spectrum of skull weights. Unfortunately, not enough data was obtained from the software for this analysis. The coefficient of variation for the four models showed no positive correlation with weight for the standard and bone algorithms with 256HU.

Another limitation is that it was not possible to measure the registration error raised by the software default registration procedure. Therefore, this unknown error is propagated to the measured mismatch between our two models. The results reported here are also limited because they are an indicator of this particular moment in time. The data reported here was based on CT scanning technology with a minimum through plane resolution of 0.625mm. Future technology will have an impact on this CT scanning threshold. Although the current results indicate a highly accurate procedure for the use of canine skull CT scans for rapid prototyping in veterinary medicine, improved procedures will be developed. Further, the model mismatch error data provided herein should be compared with the resolution of the eventual 3-D printers as the error may be within the output parameters.

The results of this study establish an efficient and accurate basis for 3-D patient-specific rapid prototyping using routine procedures. Rapid prototyping via 3-D printing has been explored in human medical fields but has only recently begun to be explored in veterinary medicine. It promises a surgeon-independent and a patient-specific approach in the veterinary neurosurgical field that could significantly shorten anesthesia times. It offers to revolutionize veterinary medical education and animal use for research through accurate anatomically detailed models. To capitalize on these opportunities, protocols must be established in the translation of veterinary medical imaging to 3-D printing. Future research should continue to establish an optimal CT imaging protocol and 3-D modelling process in veterinary medicine. The process for creating accurate 3-D skulls will have immediate clinical impact in terms of surgical planning and patient-specific implant creation. For example, the design of patient-specific cutting guides would ensure exact drilling margins compared to free-hand drilling. Both patient-specific cutting guides and implants are expected to reduce the duration of anesthesia and the risk of human error. The data obtained from this study has formed the basis for future developments in personalized medicine, patient-specific surgery, and veterinary medical education enhancement.

## Supporting information

S1 Table Table of means and standard deviations for each skull under each CT acquisition algorithm (bone vs standard) and at each software threshold setting (226HU and -650HU), including skull weights. (XLSX)
